# Supplementary material for: A Likelihood Approach for Real-Time Calibration of Stochastic Compartmental Epidemic Models
Source: PLoS Comput Biol. 2017 Jan 17;13(1):e1005257. doi: 10.1371/journal.pcbi.1005257 (PMC5240920; doi:10.1371/journal.pcbi.1005257)
Supplement: S1 File — (TAR.GZ) [file pcbi.1005257.s014.tar.gz › HSPH_Online-SI-Revision/IPoi09/n10-extreme/n10-extreme_BENCH2_table8.pdf]

1	newly 3 weeks spec	33.	31.0645	{15., 50.}	50.3421	0.228416	newly 3 weeks cumul	135.	148.984	{69., 265.}	48.4468	0.18076	newly 5 weeks spec	7.	14.1129	{6., 25.}	109.447	0.263129	newly 5 weeks cumul	159.	184.774	{85., 324.}	49.3406	0.17225	newly 50 weeks cumul	163.	214.677	{97., 391.}	55.6303	0.172471
2	newly 3 weeks spec	108.	144.483	{61., 170.}	59.4907	0.173442	newly 3 weeks cumul	418.	554.333	{263., 652.}	55.9809	0.166736	newly 5 weeks spec	44.	88.1833	{32., 106.}	110.72	0.236902	newly 5 weeks cumul	535.	754.683	{344., 886.}	61.9969	0.172304	newly 46 weeks cumul	683.	1044.73	{450., 1212.}	71.4885	0.182478
3	newly 3 weeks spec	12.	15.4426	{6., 28.}	64.4809	0.241579	newly 3 weeks cumul	49.	79.4262	{32., 133.}	78.4209	0.214096	newly 5 weeks spec	3.	5.52459	{1., 11.}	113.661	0.34327	newly 5 weeks cumul	60.	93.5902	{40., 166.}	73.4153	0.203486	newly 52 weeks cumul	67.	102.262	{43., 185.}	71.9109	0.203699
4	newly 3 weeks spec	57.	92.0656	{38., 182.}	77.1067	0.202982	newly 3 weeks cumul	214.	380.197	{168., 792.}	85.7209	0.210531	newly 5 weeks spec	34.	51.7213	{20., 118.}	74.3009	0.205615	newly 5 weeks cumul	292.	500.885	{236., 1050.}	80.9117	0.200674	newly 46 weeks cumul	396.	651.967	{296., 1426.}	76.511	0.195409
5	newly 3 weeks spec	61.	35.1129	{15., 57.}	61.1052	0.387749	newly 3 weeks cumul	281.	153.548	{65., 254.}	60.6015	0.395472	newly 5 weeks spec	29.	17.5	{8., 37.}	58.2314	0.370816	newly 5 weeks cumul	366.	195.065	{84., 334.}	61.0876	0.403497	newly 49 weeks cumul	445.	235.	{97., 413.}	61.3048	0.407863
6	newly 3 weeks spec	52.	72.5161	{36., 135.}	58.3747	0.173066	newly 3 weeks cumul	218.	331.113	{160., 591.}	64.4051	0.177797	newly 5 weeks spec	24.	33.5968	{14., 71.}	62.164	0.195904	newly 5 weeks cumul	283.	412.855	{197., 754.}	60.4981	0.171702	newly 50 weeks cumul	342.	491.952	{244., 954.}	59.2011	0.169403
7	newly 3 weeks spec	45.	30.6452	{15., 43.}	56.6308	0.315856	newly 3 weeks cumul	222.	146.371	{76., 198.}	55.5725	0.311051	newly 5 weeks spec	26.	13.0645	{5., 23.}	62.9032	0.431897	newly 5 weeks cumul	276.	179.177	{91., 247.}	55.6861	0.315032	newly 51 weeks cumul	350.	205.161	{102., 301.}	58.7189	0.355313
8	newly 3 weeks spec	74.	92.0656	{38., 182.}	57.5543	0.192772	newly 3 weeks cumul	355.	380.197	{168., 792.}	50.6534	0.194554	newly 5 weeks spec	40.	51.7213	{20., 118.}	61.9262	0.199777	newly 5 weeks cumul	452.	500.885	{236., 1050.}	51.5233	0.190029	newly 48 weeks cumul	577.	651.967	{296., 1426.}	51.9675	0.188402
9	newly 3 weeks spec	75.	73.0806	{36., 116.}	46.5161	0.198524	newly 3 weeks cumul	323.	307.903	{156., 487.}	45.4409	0.197109	newly 5 weeks spec	31.	38.4677	{17., 64.}	53.7461	0.176733	newly 5 weeks cumul	408.	399.548	{199., 644.}	45.8887	0.193622	newly 48 weeks cumul	485.	502.048	{246., 829.}	46.8341	0.186288
10	newly 3 weeks spec	97.	92.0656	{38., 182.}	52.6449	0.234723	newly 3 weeks cumul	429.	380.197	{168., 792.}	50.705	0.236127	newly 5 weeks spec	65.	51.7213	{20., 118.}	55.4351	0.288402	newly 5 weeks cumul	590.	500.885	{236., 1050.}	52.0422	0.251312	newly 48 weeks cumul	747.	651.967	{296., 1426.}	51.7063	0.244721
11	newly 3 weeks spec	96.	159.121	{55., 284.}	92.403	0.248877	newly 3 weeks cumul	365.	576.81	{217., 1037.}	82.9523	0.23904	newly 5 weeks spec	43.	107.603	{35., 166.}	161.307	0.30611	newly 5 weeks cumul	474.	814.069	{295., 1403.}	93.6345	0.246017	newly 43 weeks cumul	637.	1222.83	{413., 2054.}	112.25	0.260266
12	newly 3 weeks spec	56.	162.787	{73., 353.}	190.691	0.387327	newly 3 weeks cumul	258.	658.623	{316., 1350.}	155.407	0.338603	newly 5 weeks spec	30.	92.0656	{38., 182.}	207.869	0.400242	newly 5 weeks cumul	326.	876.033	{414., 1789.}	168.852	0.357431	newly 48 weeks cumul	370.	1147.8	{525., 2423.}	210.217	0.41579
13	newly 3 weeks spec	46.	145.365	{68., 292.}	216.011	0.429274	newly 3 weeks cumul	235.	567.413	{271., 1017.}	141.722	0.317242	newly 5 weeks spec	40.	87.7143	{40., 167.}	122.619	0.279616	newly 5 weeks cumul	321.	769.587	{361., 1413.}	140.38	0.313219	newly 44 weeks cumul	406.	1065.38	{502., 1901.}	162.409	0.346093
14	newly 3 weeks spec	50.	48.5161	{21., 85.}	53.4194	0.230199	newly 3 weeks cumul	229.	216.79	{98., 353.}	52.8032	0.227979	newly 5 weeks spec	25.	24.0161	{11., 42.}	54.2581	0.23644	newly 5 weeks cumul	291.	275.919	{126., 461.}	52.7159	0.226103	newly 49 weeks cumul	352.	333.355	{149., 577.}	52.4194	0.226078
15	newly 3 weeks spec	41.	80.8852	{38., 162.}	101.04	0.217449	newly 3 weeks cumul	175.	331.328	{168., 733.}	92.3466	0.211095	newly 5 weeks spec	20.	46.5738	{19., 91.}	136.639	0.288087	newly 5 weeks cumul	229.	440.934	{224., 970.}	95.2395	0.214371	newly 46 weeks cumul	253.	576.689	{289., 1215.}	128.135	0.274486
16	newly 3 weeks spec	68.	72.3607	{37., 129.}	47.9749	0.185741	newly 3 weeks cumul	284.	325.41	{155., 586.}	45.0358	0.159106	newly 5 weeks spec	35.	36.7869	{18., 74.}	45.6674	0.179764	newly 5 weeks cumul	368.	414.574	{212., 751.}	44.5162	0.15878	newly 49 weeks cumul	425.	500.869	{248., 894.}	46.2102	0.157271
17	newly 3 weeks spec	58.	45.4032	{23., 64.}	51.0845	0.257313	newly 3 weeks cumul	228.	219.694	{115., 312.}	45.5362	0.185111	newly 5 weeks spec	27.	19.7419	{8., 29.}	51.2545	0.29073	newly 5 weeks cumul	296.	270.081	{138., 384.}	46.5072	0.199581	newly 51 weeks cumul	355.	309.129	{155., 453.}	48.3689	0.217155
18	newly 3 weeks spec	58.	69.9194	{30., 104.}	56.2013	0.191122	newly 3 weeks cumul	249.	305.645	{142., 495.}	56.2379	0.182936	newly 5 weeks spec	33.	35.1129	{15., 57.}	54.4966	0.208469	newly 5 weeks cumul	331.	389.274	{177., 645.}	54.8533	0.186915	newly 49 weeks cumul	406.	470.726	{215., 804.}	54.4454	0.188667
19	newly 3 weeks spec	19.	45.4032	{23., 64.}	141.851	0.307422	newly 3 weeks cumul	129.	219.694	{115., 312.}	76.2816	0.184155	newly 5 weeks spec	10.	19.7419	{8., 29.}	109.032	0.267489	newly 5 weeks cumul	154.	270.081	{138., 384.}	80.2576	0.190977	newly 51 weeks cumul	172.	309.129	{155., 453.}	84.3398	0.198493
20	newly 3 weeks spec	82.	162.787	{73., 353.}	102.879	0.242849	newly 3 weeks cumul	349.	658.623	{316., 1350.}	93.2172	0.228496	newly 5 weeks spec	51.	92.0656	{38., 182.}	91.6426	0.225472	newly 5 weeks cumul	464.	876.033	{414., 1789.}	93.9161	0.228461	newly 48 weeks cumul	599.	1147.8	{525., 2423.}	96.9512	0.23283
21	newly 3 weeks spec	71.	114.117	{49., 179.}	77.5352	0.188545	newly 3 weeks cumul	319.	446.167	{201., 727.}	63.1557	0.176134	newly 5 weeks spec	24.	67.8333	{27., 111.}	184.167	0.332583	newly 5 weeks cumul	400.	603.35	{256., 965.}	70.3208	0.18135	newly 45 weeks cumul	509.	827.9	{337., 1275.}	79.9607	0.192523
22	newly 3 weeks spec	15.	26.1833	{10., 44.}	85.8889	0.22819	newly 3 weeks cumul	86.	138.283	{64., 218.}	72.3837	0.198574	newly 5 weeks spec	10.	10.9833	{4., 19.}	54.1667	0.239482	newly 5 weeks cumul	114.	165.567	{82., 263.}	61.1988	0.180926	newly 52 weeks cumul	122.	183.817	{84., 292.}	65.2049	0.187933
23	newly 3 weeks spec	49.	92.0656	{38., 182.}	97.725	0.235446	newly 3 weeks cumul	175.	380.197	{168., 792.}	120.534	0.27234	newly 5 weeks spec	26.	51.7213	{20., 118.}	107.377	0.245124	newly 5 weeks cumul	236.	500.885	{236., 1050.}	115.866	0.262441	newly 47 weeks cumul	286.	651.967	{296., 1426.}	130.769	0.28797
24	newly 3 weeks spec	62.	83.4426	{40., 128.}	60.2856	0.185626	newly 3 weeks cumul	297.	334.967	{165., 534.}	50.5934	0.184753	newly 5 weeks spec	38.	47.9836	{19., 83.}	61.7343	0.203652	newly 5 weeks cumul	391.	447.951	{214., 718.}	52.3081	0.186887	newly 45 weeks cumul	543.	602.164	{292., 942.}	52.6009	0.194611
25	newly 3 weeks spec	17.	98.7213	{45., 176.}	480.714	0.684799	newly 3 weeks cumul	104.	418.787	{188., 752.}	302.68	0.53351	newly 5 weeks spec	9.	53.8361	{22., 84.}	498.179	0.683114	newly 5 weeks cumul	127.	544.23	{252., 957.}	328.527	0.557822	newly 48 weeks cumul	139.	680.934	{314., 1219.}	389.881	0.613808
26	newly 3 weeks spec	8.	27.5323	{11., 51.}	244.153	0.459571	newly 3 weeks cumul	30.	133.032	{66., 238.}	343.441	0.580682	newly 5 weeks spec	1.	11.7258	{4., 20.}	1072.58	0.967365	newly 5 weeks cumul	36.	161.935	{81., 279.}	349.821	0.587131	newly 51 weeks cumul	40.	181.774	{88., 310.}	354.435	0.589869
27	newly 3 weeks spec	65.	89.5	{37., 156.}	59.9256	0.186799	newly 3 weeks cumul	303.	398.258	{182., 674.}	55.4775	0.178657	newly 5 weeks spec	45.	45.2903	{20., 78.}	52.043	0.223273	newly 5 weeks cumul	394.	509.548	{230., 871.}	55.3299	0.18145	newly 49 weeks cumul	475.	611.984	{278., 1021.}	55.5076	0.182713
28	newly 3 weeks spec	25.	16.3	{6., 25.}	48.1333	0.307712	newly 3 weeks cumul	120.	85.8667	{38., 143.}	48.5833	0.27746	newly 5 weeks spec	9.	7.15	{2., 13.}	57.963	0.367536	newly 5 weeks cumul	146.	104.	{49., 175.}	48.5845	0.28032	newly 52 weeks cumul	160.	115.1	{49., 186.}	48.5	0.279368
29	newly 3 weeks spec	36.	125.344	{59., 259.}	248.179	0.45848	newly 3 weeks cumul	170.	504.574	{226., 1037.}	196.808	0.396609	newly 5 weeks spec	12.	68.9672	{33., 140.}	474.727	0.669872	newly 5 weeks cumul	201.	665.607	{305., 1357.}	231.148	0.441472	newly 47 weeks cumul	219.	868.41	{384., 1851.}	296.534	0.517547
30	newly 3 weeks spec	50.	92.0656	{38., 182.}	94.5574	0.230125	newly 3 weeks cumul	199.	380.197	{168., 792.}	97.1826	0.231492	newly 5 weeks spec	23.	51.7213	{20., 118.}	129.437	0.278074	newly 5 weeks cumul	254.	500.885	{236., 1050.}	102.517	0.239283	newly 47 weeks cumul	304.	651.967	{296., 1426.}	118.41	0.267292
31	newly 3 weeks spec	53.	72.5161	{36., 135.}	56.969	0.171732	newly 3 weeks cumul	252.	331.113	{160., 591.}	52.6562	0.163281	newly 5 weeks spec	36.	33.5968	{14., 71.}	47.1774	0.222333	newly 5 weeks cumul	334.	412.855	{197., 754.}	49.5123	0.163005	newly 50 weeks cumul	377.	491.952	{244., 954.}	52.0792	0.161887
32	newly 3 weeks spec	76.	159.121	{55., 284.}	125.522	0.283134	newly 3 weeks cumul	281.	576.81	{217., 1037.}	117.002	0.274832	newly 5 weeks spec	36.	107.603	{35., 166.}	205.029	0.355438	newly 5 weeks cumul	374.	814.069	{295., 1403.}	128.195	0.283254	newly 42 weeks cumul	533.	1222.83	{413., 2054.}	141.586	0.289969
33	newly 3 weeks spec	8.	30.6452	{15., 43.}	284.274	0.494343	newly 3 weeks cumul	52.	146.371	{76., 198.}	181.793	0.369756	newly 5 weeks spec	2.	13.0645	{5., 23.}	553.226	0.711445	newly 5 weeks cumul	59.	179.177	{91., 247.}	203.745	0.402572	newly 50 weeks cumul	62.	205.161	{102., 301.}	230.905	0.436962
34	newly 3 weeks spec	8.	8.63934	{3., 19.}	58.8115	0.256139	newly 3 weeks cumul	61.	48.1311	{21., 86.}	49.6909	0.269515	newly 5 weeks spec	7.	3.14754	{0., 9.}	70.0234	0.722139	newly 5 weeks cumul	76.	56.8033	{23., 110.}	51.1864	0.287649	newly 52 weeks cumul	80.	62.3279	{25., 121.}	50.2049	0.274921
35	newly 3 weeks spec	95.	68.9672	{33., 140.}	56.2899	0.309149	newly 3 weeks cumul	347.	286.377	{136., 579.}	53.3094	0.264676	newly 5 weeks spec	52.	37.9508	{14., 91.}	56.0214	0.32108	newly 5 weeks cumul	464.	376.049	{172., 788.}	53.8051	0.271759	newly 47 weeks cumul	569.	489.18	{221., 1073.}	52.7212	0.253818
36	newly 3 weeks spec	110.	145.365	{68., 292.}	56.4502	0.179149	newly 3 weeks cumul	389.	567.413	{271., 1017.}	61.9415	0.180159	newly 5 weeks spec	47.	87.7143	{40., 167.}	94.4613	0.232922	newly 5 weeks cumul	515.	769.587	{361., 1413.}	64.9684	0.184887	newly 45 weeks cumul	717.	1065.38	{502., 1901.}	65.4668	0.186178
37	newly 3 weeks spec	60.	100.403	{45., 166.}	78.1452	0.201591	newly 3 weeks cumul	245.	418.339	{209., 690.}	78.2554	0.200663	newly 5 weeks spec	22.	53.1774	{21., 91.}	144.648	0.3144	newly 5 weeks cumul	316.	544.597	{268., 903.}	79.4355	0.201814	newly 48 weeks cumul	370.	685.565	{336., 1152.}	90.449	0.221033
38	newly 3 weeks spec	10.	27.5323	{11., 51.}	175.645	0.364137	newly 3 weeks cumul	32.	133.032	{66., 238.}	315.726	0.552653	newly 5 weeks spec	1.	11.7258	{4., 20.}	1072.58	0.967365	newly 5 weeks cumul	36.	161.935	{81., 279.}	349.821	0.587131	newly 51 weeks cumul	37.	181.774	{88., 310.}	391.282	0.623727
39	newly 3 weeks spec	14.	27.5323	{11., 51.}	104.724	0.256955	newly 3 weeks cumul	85.	133.032	{66., 238.}	66.5275	0.179112	newly 5 weeks spec	6.	11.7258	{4., 20.}	113.71	0.30774	newly 5 weeks cumul	101.	161.935	{81., 279.}	69.4986	0.184608	newly 51 weeks cumul	112.	181.774	{88., 310.}	72.1774	0.192167
40	newly 3 weeks spec	73.	100.403	{45., 166.}	58.7494	0.175029	newly 3 weeks cumul	282.	418.339	{209., 690.}	62.9318	0.176989	newly 5 weeks spec	30.	53.1774	{21., 91.}	88.871	0.226747	newly 5 weeks cumul	357.	544.597	{268., 903.}	65.876	0.181245	newly 48 weeks cumul	439.	685.565	{336., 1152.}	68.7009	0.185065
41	newly 3 weeks spec	66.	92.0656	{38., 182.}	64.1331	0.191248	newly 3 weeks cumul	240.	380.197	{168., 792.}	70.888	0.184952	newly 5 weeks spec	31.	51.7213	{20., 118.}	83.7652	0.215109	newly 5 weeks cumul	309.	500.885	{236., 1050.}	73.8554	0.189027	newly 47 weeks cumul	381.	651.967	{296., 1426.}	81.4122	0.203456
42	newly 3 weeks spec	69.	159.121	{55., 284.}	142.604	0.301144	newly 3 weeks cumul	344.	576.81	{217., 1037.}	89.4898	0.245252	newly 5 weeks spec	67.	107.603	{35., 166.}	94.1585	0.25292	newly 5 weeks cumul	474.	814.069	{295., 1403.}	93.6345	0.246017	newly 42 weeks cumul	655.	1222.83	{413., 2054.}	108.465	0.257181
43	newly 3 weeks spec	15.	40.0806	{17., 62.}	170.86	0.357107	newly 3 weeks cumul	71.	199.355	{78., 298.}	181.917	0.363845	newly 5 weeks spec	6.	16.7419	{7., 26.}	182.796	0.364313	newly 5 weeks cumul	88.	242.016	{98., 367.}	176.191	0.355261	newly 52 weeks cumul	93.	269.532	{114., 410.}	190.652	0.375803
44	newly 3 weeks spec	89.	144.933	{51., 231.}	82.5468	0.224132	newly 3 weeks cumul	399.	545.217	{211., 929.}	62.9616	0.201625	newly 5 weeks spec	51.	94.9	{30., 147.}	102.81	0.233079	newly 5 weeks cumul	518.	758.367	{285., 1280.}	69.2986	0.204164	newly 43 weeks cumul	705.	1112.15	{416., 1743.}	80.5414	0.211161
45	newly 3 weeks spec	84.	125.344	{59., 259.}	65.7299	0.17849	newly 3 weeks cumul	323.	504.574	{226., 1037.}	68.1927	0.179994	newly 5 weeks spec	39.	68.9672	{33., 140.}	86.0866	0.208197	newly 5 weeks cumul	423.	665.607	{305., 1357.}	69.771	0.183118	newly 47 weeks cumul	539.	868.41	{384., 1851.}	73.2322	0.189851
46	newly 3 weeks spec	113.	53.3492	{21., 97.}	59.9241	0.435117	newly 3 weeks cumul	424.	208.968	{97., 376.}	58.4868	0.41224	newly 5 weeks spec	54.	31.8254	{12., 65.}	57.1723	0.382621	newly 5 weeks cumul	553.	282.683	{131., 505.}	57.8088	0.401677	newly 45 weeks cumul	782.	383.508	{169., 693.}	59.2965	0.418855
47	newly 3 weeks spec	79.	125.344	{59., 259.}	71.8614	0.186792	newly 3 weeks cumul	324.	504.574	{226., 1037.}	67.8759	0.179532	newly 5 weeks spec	43.	68.9672	{33., 140.}	73.6561	0.187928	newly 5 weeks cumul	419.	665.607	{305., 1357.}	70.797	0.184674	newly 47 weeks cumul	531.	868.41	{384., 1851.}	75.0023	0.192725
48	newly 3 weeks spec	58.	72.5161	{36., 135.}	52.1135	0.171929	newly 3 weeks cumul	234.	331.113	{160., 591.}	57.8784	0.168222	newly 5 weeks spec	22.	33.5968	{14., 71.}	70.6012	0.207524	newly 5 weeks cumul	292.	412.855	{197., 754.}	57.6502	0.167364	newly 50 weeks cumul	344.	491.952	{244., 954.}	58.6694	0.168586
49	newly 3 weeks spec	22.	45.4032	{23., 64.}	111.364	0.255357	newly 3 weeks cumul	126.	219.694	{115., 312.}	79.5827	0.190531	newly 5 weeks spec	10.	19.7419	{8., 29.}	109.032	0.267489	newly 5 weeks cumul	151.	270.081	{138., 384.}	83.2621	0.19704	newly 51 weeks cumul	165.	309.129	{155., 453.}	91.0068	0.211636
50	newly 3 weeks spec	52.	134.419	{63., 229.}	158.499	0.345005	newly 3 weeks cumul	223.	562.145	{280., 976.}	152.083	0.338123	newly 5 weeks spec	29.	73.0806	{36., 116.}	153.115	0.332041	newly 5 weeks cumul	274.	735.629	{363., 1242.}	168.478	0.36344	newly 49 weeks cumul	323.	929.774	{452., 1584.}	187.856	0.3906
